# Hæmorrhage after Operative Cure of Intussusception

**Published:** 1908-03-28

**Authors:** 


### Diseases of Children.

### HÆMORRHAGE AFTER OPERATIVE CURE OF INTUSSUSCEPTION.

Let it be supposed that an infant of nine months or a year old has had all the classical symptoms of intussusception, and has been operated upon within twelve hours of the beginning of those symptoms, life being thereby saved for the time being. The great majority of these little patients do very well if they have survived the first forty-eight hours after the operation. They nearly all "run a temperature" for several days without any apparent danger resulting. There is, however, one untoward symptom which now and again presents itself a week, ten days, or a fortnight after the successful operation, and that is the recurrence of the passage of blood and mucus per rectum.

Little or nothing about this is to be found in most text-books, and when a case of the kind presents itself it is difficult to know what is the best thing to be done. The infant has quite recovered from the original operation, and its subsequent pyrexia; the abdominal wound has healed up cleanly and well; the patient has been taking ordinary feeds of milk and barley water without the slightest sign of sickness; everything has seemed to be progressing in an absolutely favourable way. All of a sudden the child ceases to take its food as well as it has been doing hitherto; it may perhaps be a little sick; it passes no proper motion, but in the stead of fæces there is definite blood and a little mucus upon the diapers. The case is anxiously watched, and the blood and mucus are repeated several times, and the child begins to go downhill.

The first idea that naturally enters one's mind is that the intussusception has recurred. The abdomen is carefully palpated for a lump. None can be felt anywhere away from the operation scar, but at the site of the latter it is difficult to feel anything definitely, and one is in doubt as to whether the resistance there is simply and solely due to the slight matting beneath the scar, or whether there is really a soft lump in that region as well. The probability is that a second operation will be performed, because it will be thought that one cannot be sure that the intussusception has not recurred, and because, if it has, the less delay there is the better will be the patient's chances of recovery.

It is, of course, possible that a recurrence of the intussusception will be found very occasionally; but, to judge from a series of consecutive cases, this is exceedingly unlikely. At any rate, the number of cases in which this recurrence of bowel hæmorrhage occurs, small though it is, is yet much greater than is the number in which there is recurrence of the intussusception.

Too often, to one's dismay, nothing is found to account for the symptom, and the infant dies within twenty-four hours afterwards. If, on the other hand, nothing operative is done, the child being treated merely on the lines of an acute entero-colitis, death occurs almost as certainly. In either case, at the post-mortem examination, nothing whatever is visible to the naked eye to account for the blood and mucus being passed per rectum.

The probability is that the infant's best chance of recovery is to adopt expectant treatment, and not to act as though there had been a recurrence of the intussusception unless a definite lump can be felt in the abdomen. The precise nature of the mischief is quite obscure, although it is a very distressing thing to have a child dying in this way when everything had seemed to be going perfectly well. It is a little surprising that the books do not say more about the condition, for it is distinctly of importance to recognise that recurrence of the symptoms of intussusception during convalescence from operation by no means implies that there has been recurrence of the intussusception itself.

